# Fake news and fake research: Why meta‐research matters more than ever

**DOI:** 10.1111/jpc.15237

**Published:** 2020-10-21

**Authors:** Richard G McGee, Amanda C Dawson

**Affiliations:** ^1^ The Central Coast Clinical School, School of Medicine and Public Health The University of Newcastle Newcastle New South Wales Australia; ^2^ Department of Paediatrics Gosford Hospital Newcastle New South Wales Australia; ^3^ Department of Surgery Gosford Hospital Gosford New South Wales Australia

**Keywords:** evidence‐based medicine, meta‐research, research method, scientific method

## Abstract

Research is in a crisis of credibility, and this is to the peril of all paediatricians. Billions of dollars are being wasted each year because research is not planned, badly conducted or poorly reported, and this is on a background of rapidly reducing research budgets. How can paediatricians, families and patients make informed treatment choices if the evidence base is absent or not trustworthy? This article discusses why meta‐research now matters more than ever, how it can help solve this crisis of credibility and how this should lead to more efficient and effective clinical care. The field of meta‐research or research‐on‐research is the ultimate big picture approach to identifying and solving issues of bias, error, misconduct and waste in research. Meta‐researchers value authenticity over aesthetics and quality over quantity. The utility of meta‐research does not rely on accusations or critical assessments of individual research, but through highlighting where and how the scientific method and research standards across all fields can be improved. Meta‐researchers study, analyse and critique the research pathway, focusing on elements such as methods (how to conduct), evaluation (how to test), reporting (how to communicate), reproducibility (how to verify) and incentives (how to reward). In the current climate it is now more critical than ever that we make use of meta‐research and prioritise high‐quality high‐impact research, ultimately leading to improved patient outcomes.

## Fake News and Fake Research

‘Fake news’ and ‘fake research’ are realities of the modern world and part of the reason scientists, researchers and clinicians are facing a crisis of credibility. Approximately 2% of researchers admit to deliberate research misconduct and 33% admit to questionable research practices.^1^ Regardless of misconduct, researcher John Ionnaidis has established that because of bias and chance most research findings are either not useful or false.^2^ Furthermore, in 2009 Chalmers and Glasziou showed that because of a lack of planning and issues with reporting about 85% of more than US$100 billion/year spent on medical research globally was being wasted.^3^ More recently, the COVID‐19 pandemic has brought a new deluge of papers, many of questionable quality, a situation described by the United Nations as an ‘infodemic of misinformation’.^4^, ^5^ The COVID‐19 pandemic has also led to reductions in research funding and so it is now more critical than ever that we prioritise high‐quality high‐impact research, which is the primary goal of meta‐research.^6^ Improving the quality of the evidence base should lead to more efficient and effective clinical care, improved outcomes and increased credibility. These are lofty objectives but reflect the importance of optimising the scientific method.

There are two common misconceptions about meta‐research. One is that meta‐researchers critique individual research studies. In reality, meta‐researchers usually take a bird's‐eye view to the way research is performed across broad areas. While meta‐researchers assess research standards, importantly, they also suggest how to improve research standards. Trial registration, reporting guidelines and assessments of bias are examples of quality‐improvement efforts, which have come about because of previous meta‐research studies. The second misconception is that meta‐analyses are sometimes confused with meta‐research. However, meta‐analyses are a statistical technique usually conducted within a systematic review seeking to answer a specific clinical query, while meta‐research is a field of research, which focuses on keeping research robust and relevant.

We now discuss the role of meta‐research in paediatrics according to the key steps of the research process.

## Research Prioritisation

We can reduce wastage of research resources by matching research efforts to disease burden and areas of clinical need.^7^ Meta‐research helps identify and highlight specific areas of disconnect by reviewing published literature across different disciplines. One example was a review of ongoing and published paediatric drug therapy trials in the European Union (EU) in 2008, which found that only four of the 25 European Medicines Agency priority items were being studied.^8^ This is despite the European Medicines Agency being the agency responsible for the scientific evaluation, supervision and safety monitoring of medicines across the EU.^9^ Beyond the EU, on a global scale, meta‐research studies have also shown a significant disconnect between areas of paediatric disease burden and the areas where clinical trials are being conducted.^10^, ^11^ Unfortunately, the discrepancy between disease burden and research effort is more pronounced in low‐ and middle‐income countries, and these countries already suffer greater disease burden.^12^ We repeatedly see this disconnect across many disciplines, including paediatric cardiology,^13^ critical care,^14^ oncology^15^ and primary care.^16^ To address these problems, researchers have created evidence‐based research agendas.^17^ Globally discrepancies also exist between disease burden and research funding.^18^ But some funding agencies are now starting to prioritise research according to disease burden.^19^ Future meta‐researchers should test the ability of priority‐driven research agendas to improve equity and reduce wasted resources.

## Methods

Assessing and improving study design and methods is a key step in improving research standards. It is now relatively routine to consider how to reduce bias and improve the conduct of a research study, but this is because of the many fundamental meta‐research articles, which provided empirical evidence for the impact of bias on outcomes.^20^, ^21^, ^22^ Researchers and organisations have introduced various reforms to minimise bias in studies and improve research reporting. For example, in 2005 the International Committee of Medical Journal Editors (ICMJE) introduced trial registration to reduce reporting bias and data dredging.^23^ While in 2010, researchers published an extension of the Consolidated Standards Of Reporting Trials (CONSORT) statement specific to paediatrics, termed CONSORT‐C, to improve the completeness of paediatric trial reporting.^24^ Assessments of the paediatric literature have shown variable impact from these interventions. A study of approximately 600 paediatric randomised trials published between 2007 and 2012 showed an improvement in the use of allocation concealment (hiding the method of sorting trial participants into treatment groups) and trial registration, perhaps reflecting the introduction of the ICMJE statement and CONSORT‐C extension amongst other developments.^25^

Implementing blinding is another key step in the research process as blinding reduces performance and detection bias. However, researchers often consider it difficult to implement blinding.^26^ A critical assessment in neonatology of almost 2000 randomised trials in 2019 showed that, despite a steady improvement in the overall quality of trials since the 1950s, there was no clear improvement in implementing blinding.^27^ Other disciplines, for example surgery, have also found it difficult to implement blinding but are now beginning to develop detailed guidelines to assist researchers.^28^ To date, similar guides have not been produced in paediatrics; however, groups such as STaR Child Health are working on it.^29^

## Hypothesis Testing

Meta‐research helps to improve hypothesis testing and evaluation, which are cornerstones of research practice. In particular, it is important to ensure that research is adequately powered, to reduce the risk of type I (false positive) and type II (false negative) errors.

Meta‐research studies have shown that trials in paediatrics are often underpowered, with a high‐fragility index.^30^ A high‐fragility index means a few extra events could alter a study's result, and so we might consider the study less reliable. The STaR Child Health research group, and the CONSORT‐C extension, aim to improve reliability by assisting researchers in appropriately determining and reporting their power calculations.^24^, ^31^

Ensuring tests of statistical significance are properly interpreted and reported is another fundamental part of meta‐research. Researchers may not reach statistical significance for a trial's primary outcome if the trial is underpowered from low recruitment, funding difficulties and/or premature termination.^32^ The consequence is that this makes the research difficult to interpret and publish.^33^ Some researchers even engage in the dubious practice of ‘p‐hacking’ to reach statistical significance.^34^ Such issues have led to a search for alternative ways of expressing statistical significance.^35^ As future research emerges, we need to continue to refine the best solutions for evaluating hypotheses.

## Reporting and Publication

The standards and expectations of research reporting have changed over time, but it has proven unexpectedly difficult to improve the standard of reporting within the paediatric literature. While the ICMJE statement on trial registration came out in 2005, to date the uptake has been poor in both adult and paediatric medicine.^36^ The CONSORT statement and other reporting guidelines, promoted by groups such as the Enhancing the QUAlity and Transparency Of health Research (EQUATOR) network, have sought to improve the completeness of trial reporting. However, deficiencies in reporting are still found in areas such as study protocols,^37^ consent and recruitment,^38^ description of the primary outcome^39^ and reports to data monitoring committees.^40^ Reporting guidelines are used inappropriately and authors have even been found to ‘spin’ the wording of articles to distort the interpretation of results.^41^, ^42^ Improving the wording, content and clarity of research reports is an area of active development and a range of tools and guidelines continue to be developed.^43^

## Reproducibility

One of the many reasons that research has been viewed as having a crisis of credibility has been the inability to verify and reproduce research findings.^44^ Meta‐researchers have shown that early initial studies, and studies stopped prematurely, are likely to overestimate treatment effects and stifle future research.^45^ In addition, the failure of authors to provide accessible datasets hinders study verification and reassessment. Studies highlighting the need for transparency and reproducibility have been taken on board by funding agencies, such as the National Institutes of Health, who now have an open data policy.^46^ Paediatric journals, to date, have not pushed as strongly for open data,^47^ despite evidence showing the benefits of such a policy.^48^ Other methods of incentivising researchers to provide their data need to be considered.

## Incentives

For many researchers, career advancement and economic reward are tightly linked to their publication track record with a traditional emphasis on the quantity over quality of publications produced. The desire to publish a large quantity of work for reward may conflict with the time commitment required to produce high quality research. We need to address this conflict of interest between incentives and research standards by rethinking how we reward researchers. The Hong Kong Principles were recently developed as part of the 6th World Conference on Research Integrity and aim to ensure that researchers are explicitly recognised and rewarded for behaviours that strengthen research integrity.^49^ Other initiatives, such as the San Francisco Declaration on Research Assessment (DORA) and The Leiden Manifesto,^50^, ^51^ which promote assessment of research quality when considering decisions around investments, allocation of resources, promotions and recruitment, are now starting to gain greater acceptance within academia.^52^

## Summary

Meta‐researchers approach the scientific process from a bird's‐eye view and highlight key steps where improvements can be made, ensuring research remains robust, relevant and credible. As paediatricians we should fight against fake research, poor‐quality research and low‐value research. Therefore, we all have a vested interest in meta‐research and improving the evidence base so we can deliver best care for patients. This is why meta‐research matters more than ever.
